# *Bacillus velezensis* RF2 Rescued from Citrus Phyllosphere: Dual Mechanisms and Broad-Spectrum Activity for Controlling Citrus Bacterial Canker

**DOI:** 10.3390/microorganisms14010121

**Published:** 2026-01-06

**Authors:** Rui-Fang Luo, Si-Yu Zhang, Ya-Xiao Wu, Zi-Yi Jiao, Min-Li Bao, Yu-Ting Lan, Ting-Ting Zhang, Ru-Yu Zeng, Abdulhamid Yusuf, Yun-Zeng Zhang, Min Li, Shuo Duan

**Affiliations:** 1 Jiangxi Provincial Key Laboratory of Pest and Disease Control of Featured Horticultural Plants, Gannan Normal University, Ganzhou 341003, China13386772386@163.com (Y.-T.L.); 13033219954@163.com (T.-T.Z.); 19507971834@163.com (R.-Y.Z.); 2 Joint International Research Laboratory of Agriculture and Agri-Product Safety of the Ministry of Education, Yangzhou University, Yangzhou 225009, Chinayzzhang@yzu.edu.cn (Y.-Z.Z.)

**Keywords:** citrus bacterial canker, phyllosphere microorganisms, ‘Cry for Help’, *Bacillus velezensis* RF2

## Abstract

Citrus bacterial canker (CBC), caused by *Xanthomonas citri* subsp. *citri* (*Xcc*), threatens citrus production worldwide. Long-term dependence on copper-based bactericides not only poses environmental risks but also accelerates the emergence of copper-resistant *Xcc* strains. To develop safe and efficient alternative control strategies, 72 bacterial strains were isolated from the phyllosphere of citrus plants naturally infected by CBC and identified by 16S rRNA sequencing. Using an *Xcc*-GFP-based screening method, we systematically screened a highly effective strain, which was identified as *Bacillus velezensis* RF2 (Bv-RF2). Both inhibition zone assays and bioactivity tests of the crude methanolic extract of Bv-RF2 demonstrated stable antibacterial activity under UV irradiation, protease treatment, high temperature, and across a wide pH range. Whole-genome sequencing and antiSMASH analysis revealed multiple predicted NRPS/PKS-type biosynthetic gene clusters (BGCs). Together with metabolomic profiling, these data provide hypotheses for candidate metabolites that may contribute to antagonism. Bv-RF2 was associated with the induction of *PR* gene expression in immune-related pathways implicated in CBC responses. In sweet orange leaves, Bv-RF2 infiltration was associated with transient induction of defense-related (PR) genes, consistent with an ISR-like, priming-related response. In addition, Bv-RF2 inhibited the growth of fungal pathogens associated with citrus anthracnose and brown spot in vitro, indicating broad inhibitory potential under the tested conditions. Collectively, Bv-RF2 represents a promising candidate for developing environmentally friendly strategies against CBC and other citrus diseases.

## 1. Introduction

Citrus is one of the most widely cultivated and economically important fruit crops worldwide; however, its production is seriously constrained by diseases. Citrus bacterial canker (CBC), caused by *Xanthomonas citri* subsp. *citri* (*Xcc*), is particularly destructive in major citrus-producing regions [1,2,3]. *Xcc* infects leaves, shoots, and fruits, causing water-soaked lesions, corky eruptions, and tissue hyperplasia, which may lead to defoliation, premature fruit drop, yield losses, and reduced fruit quality [4,5,6]. CBC therefore represents a key bottleneck for sustainable citrus production.

Multiple pathogenic types are associated with citrus canker, including *Xcc*A, *Xcc*A*, *Xcc*Aw, and *X. fuscans* subsp. *aurantifolii* B and C [7,8,9,10]. Among them, the highly virulent Asian type *Xcc*A is globally dominant. After entering through stomata or wounds, *Xcc* employs a type III secretion system (T3SS) to deliver transcription activator-like effectors (TALEs) into host nuclei [4,11]. The effector PthA4 activates the susceptibility gene *CsLOB1*, thereby promoting cell overproliferation and typical canker symptoms [12,13]. Additional virulence factors, including lipopolysaccharides, xanthan gum, and biofilms, promote colonization, stress tolerance, and pathogen spread [14,15,16].

Current CBC management still relies heavily on copper-based bactericides, which primarily act through copper-mediated damage to bacterial macromolecules [17,18]. However, long-term and intensive use has led to copper accumulation in soil and water, ecological concerns, and the emergence of copper-resistant *Xcc* strains [18,19,20]. Other approaches—such as breeding resistant cultivars, quarantine, and chemical inducers of resistance—are often limited by long breeding cycles, high costs, or inconsistent performance [21]. There is an urgent need for safe, effective, and environmentally friendly alternatives.

Biological control offers a promising strategy for greener CBC management. The “Cry for Help” hypothesis proposes that plants under pathogen stress can reshape their microbiomes by recruiting beneficial microorganisms that enhance disease resistance [22,23,24]. In citrus, *Diaporthe citri* infection enriches disease-suppressive taxa (e.g., *Pantoea* asv90, *Methylobacterium* asv41), increases microbial network complexity, and enhances functions such as siderophore production and pathogen cell wall degradation, resulting in disease inhibition rates up to 65.7–88.4% [22]. Citrus leaves infected with *Xcc* may undergo similar microbiome shifts, providing a rational reservoir for mining of candidate biocontrol strains.

Targeted isolation of biocontrol bacteria from *Xcc*-infected leaves has several advantages: (i) infection may increase the relative abundance of antagonistic bacteria, improving screening efficiency [25,26]; (ii) isolates may be pre-adapted to the citrus phyllosphere and to coexistence with *Xcc*, conferring niche compatibility and competitive fitness [25,27,28]; and (iii) this approach reduces randomness and prioritizes naturally selected candidates.

Among potential biocontrol agents, *Bacillus velezensis* has attracted particular interest. It is a Gram-positive member of the *B. subtilis* species complex and is widely distributed in soil and plant-associated habitats [29,30]. *B. velezensis* strains can suppress diverse fungi, including *Colletotrichum capsici* [31], and *Xcc* [32], and can enhance systemic resistance against Tobacco mosaic virus (TMV) [33]. Their biocontrol performance is often associated with: (i) production of diverse secondary metabolites, including lipopeptides (e.g., surfactin, iturin, and fengycin), polyketides (e.g., macrolactin and bacillaene), and volatile organic compounds (e.g., 2,3-butanediol) [34,35,36]; (ii) elicitation of SAR or ISR-like responses mediated mainly by jasmonic acid (JA), ethylene (ET), and NPR1-dependent signaling [37,38,39]; and (iii) plant growth promotion via phytohormones such as indole-3-acetic acid (IAA) [40]. In addition, *Bacillus* species form stress-resistant endospores and can colonize plant tissues efficiently, supporting survival under field conditions [41,42].

In this study, guided by a “microbiome distress response under citrus canker stress” framework and an *Xcc*-GFP-based high-throughput screening method, we mined the phyllosphere of *Xcc*-infected citrus to identify effective biocontrol bacteria. We identified *B. velezensis* RF2 as a core candidate and integrated whole-genome sequencing, biosynthetic gene cluster (BGC) prediction, metabolite/extract bioactivity assays, and host response analyses to investigate its potential modes of action and broad inhibitory activity. Notably, genome-predicted BGCs provide mechanistic hypotheses; therefore, we interpret genotype–phenotype links cautiously and highlight remaining limitations and future validation needs.

## 2. Materials and Methods

### 2.1. Pathogens and Plant Materials

*Xanthomonas citri* subsp. *citri* (*Xcc*) was isolated from citrus orchards in Jiangxi Province, China, and cultured at 28 °C on Nutrient Broth (NB) or Nutrient Agar (NA) (Landbridge, Beijing, China). Where required, 50 mg/L kanamycin (Macklin, Shanghai, China) was added.

*Colletotrichum gloeosporioides* and *Alternaria alternata* were cultured on Potato Dextrose Agar (PDA) prepared from a commercially available PDA powder (Hopebiol, Qingdao, China) according to the manufacturer’s instructions and incubated under a 16 h/8 h light/dark cycle at 28/26 °C and 80% relative humidity.

‘Hamlin’ sweet orange (*Citrus sinensis* (L.) Osbeck) plants were maintained in a greenhouse under a 16 h/8 h light/dark cycle at 28/26 °C and 80% relative humidity. Fully expanded leaves (approximately one month old) were used for all assays.

### 2.2. Isolation and Identification of Citrus Phyllosphere Bacteria

Citrus plants naturally infected with *Xcc* and showing similar growth status were selected in the orchard. Leaves from the four canopy quadrants were pooled and transferred into 50 mL tubes containing sterile PBS (137 mM NaCl, 2.7 mM KCl, 2 mM KH_2_PO_4_, 8 mM Na_2_HPO_4_·12 H_2_O, pH 7.2–7.4) (Sinopharm, Beijing, China). Samples were vortexed for 15 s, and the wash solution was collected, serially diluted (10-fold), and plated on NA. After incubation at 28 °C for 24–48 h, single colonies were purified. To isolate endophytic bacteria, leaves were surface-sterilized, ground, and the homogenate was serially diluted and plated. All isolates were stored in 15% (*v*/*v*) glycerol (Xilong, Shantou, China) at −80 °C.

Genomic DNA was extracted using the HeaPure Bacterial Genomic DNA Extraction Kit (Coolaber, Beijing, China). The 16S rRNA gene was amplified using primers 27F (5′-AGAGTTTGATCCTGGCTCAG-3′) and 1492R (5′-TACGACTTAACCCCAATCGC-3′). PCR products were verified by agarose gel electrophoresis and sequenced by Genewiz (Guangzhou, China). Assembled sequences were compared against the NCBI nucleotide database.

### 2.3. Identification and Characterization of Candidate Biocontrol Strains

Candidate biocontrol strains were screened using an *Xcc*-GFP-based method as described previously [18]. Selected isolates were streaked on NA and incubated at 28 °C for 24–48 h to examine colony morphology. Gram staining was performed for microscopic observation. Genomic DNA was extracted as described above. The 16S rRNA gene was amplified and cloned into a *pEASY*-Blunt Cloning Vector, followed by transformation into *Trans1*-T1 Phage Resistant Chemically competent cells (Transgen, Beijing, China). Positive transformants were identified using primers M13F (5′-GTAAAACGACGGCCAGT-3′) and M13R (5′-CAGGAAACAGCTATGAC-3′) and sequenced (Genewiz). Assembled 16S rRNA sequences were submitted to NCBI, and a phylogenetic tree was constructed using the Neighbor-Joining method in MEGA 11 (version 11.0.18, MEGA Software Team, Tempe, AZ, USA) with 1000 bootstrap replicates.

### 2.4. Methanol Extraction and Stability Evaluation of Bv-RF2 Fermentation Products

Antimicrobial metabolites from Bv-RF2 were prepared with minor modifications to Lin et al. [43]. Glycerol stocks were streaked on NA and incubated at 28 °C. A single colony was inoculated into 100 mL NB and cultured at 28 °C with shaking at 200 rpm for 16 h (seed culture). The seed culture (10%, *v*/*v*; initial OD_600_ = 0.2–0.3) was transferred into 800 mL NB and incubated at 28 °C with shaking at 200 rpm for 48 h. Cultures were centrifuged at 10,000 rpm for 15 min at 4 °C to obtain cell-free supernatant (CFS).

For acid precipitation, the pH of the CFS was adjusted to 2.0 with 6 N HCl (Xilong, Shantou, China) and incubated at 4 °C overnight. Precipitates were collected by centrifugation (10,000 rpm, 15 min, 4 °C), washed twice with ddH_2_O (pH 2.0), resuspended in ddH_2_O, and adjusted to pH 7.0. After centrifugation, the supernatant was evaporated to dryness at 60 °C. The residue was dissolved in methanol, insoluble material was removed, and the methanol phase was evaporated at 45 °C and weighed.

The crude methanolic extract was dissolved in sterile water to 20 mg/mL and further diluted to 10, 5, 2.5, and 1 mg/mL. Antagonistic activity against *Xcc*-GFP was assessed using an agar well diffusion (“cup”) assay. For stability assays, a 1 mg/mL solution was subjected to: (i) heat treatments at 30, 45, 60, 75, 90, or 100 °C for 30 min; (ii) UV irradiation (254 nm) for 5–50 min; (iii) protease treatment with 5 μg/mL trypsin or proteinase K at 37 °C for 2 h; and (iv) pH treatments (2, 4, 7, 10, or 12) for 24 h, followed by readjustment to pH 7.0 prior to testing. 50 μL were added per well. Each treatment included three technical replicates, and the entire experiment was independently repeated three times.

### 2.5. Effects of Bv-RF2 on Sweet Orange Immune Pathways and Defense Gene Expression

Bv-RF2 was grown overnight in NB at 28 °C with shaking. Cells were harvested (12,000 rpm, 3 min), washed, and resuspended in sterile water (OD_600_ = 1.0). Bacterial suspensions were infiltrated into fully expanded leaves using a needleless syringe, with ddH_2_O as the control. Three independent biological replicates were included.

Leaf samples were collected at 0, 1, 3, 5, 7, 9, 12, and 15 days post-inoculation (dpi) for RNA extraction. Defense-related gene expression was quantified by RT-qPCR using *CsGAPDH* as the reference gene. Primer sequences are listed in Table S1.

For Evans blue (Coolaber, Beijing, China) staining, leaves were immersed in ddH_2_O and shaken at 37 °C, 200 rpm for 12 h, then incubated in 0.25% (*w*/*v*) Evans blue for 24 h. Excess dye was removed with purified water, and leaves were photographed after blotting. To enhance contrast, leaves were decolorized in boiling ethanol:glycerol (9:1, *v*/*v*) for 30 min until chlorophyll was removed, then flattened and photographed.

For relative electrolyte leakage, clean leaves were rinsed three times with ddH_2_O and immersed in 30 mL ddH_2_O, followed by shaking at 37 °C (200 rpm) for 24 h. Initial conductivity (Lt) was measured. Samples were then heated at 90 °C for 2 h to obtain total conductivity (L_0_). Relative electrolyte leakage (L) was calculated as L = (Lt/L_0_) × 100%.

### 2.6. Biocontrol Assay on Sweet Orange Leaves

An *Xcc*-GFP suspension (OD_600_ = 0.6) was mixed (1:1, *v*/*v*) with Bv-RF2 crude methanol extract at 10, 5, or 1 mg/mL. The control consisted of *Xcc*-GFP mixed with ddH_2_O. Tween-20 (0.1%, *v*/*v*) was added, and the final *Xcc*-GFP concentration was approximately 5.0 × 10^8^ CFU/mL. On the abaxial side of ‘Hamlin’ leaves (2–3 weeks after flush) from 3–4-year-old trees, 28 wounds per leaf were made using a dissecting pin. Each wound was inoculated with 2 μL of the corresponding mixture, and inoculation sites were kept moist. Three leaves were used per treatment group. Disease development was monitored, and lesion areas were quantified at 7 dpi. Data were analyzed using GraphPad Prism 10. The experiment was independently repeated three times.

### 2.7. Whole-Genome Sequencing and Secondary Metabolite Gene Cluster Analysis of Bv-RF2

Bv-RF2 genomic DNA was sequenced by Megigene (Megigene, Guangzhou, China) using both Illumina (short-insert paired-end library) and Oxford Nanopore Technologies platforms. After quality control, hybrid de novo assembly was performed with Unicycler [44]. Coding sequences were predicted using Glimmer3 [45] or Prodigal [46] (for GC > 70%). tRNAs were predicted using tRNAscan-SE v1.4 [47], rRNAs using rRNAmmer v1.2 [48], and sRNAs by comparison against Rfam [49] followed by CMsearch filtering [50]. Repetitive and tandem repeats were identified using RepeatMasker v4.1.2-p1 and TRF v4.09 [51], respectively. Functional annotation was performed using BLAST (https://blast.ncbi.nlm.nih.gov/Blast.cgi) and DIAMOND (version 2.1.9, Buchfink B, Xie C, Huson DH, Tübingen, Germany). Secondary metabolite biosynthetic gene clusters (BGCs) were predicted using antiSMASH (https://antismash.secondarymetabolites.org/#!/start) under the strict “known cluster detection” setting to infer BGC types, genomic locations, and putative products.

### 2.8. Assessment of Representative Metabolite Activity

Log-phase *Xcc*-GFP cells were scraped from NA plates, washed three times with PBS, and resuspended to OD_600_ = 1.0. Aliquots (0.1 mL) were mixed with 10 mL of molten 1.2% NA, poured into Petri dishes, and allowed to solidify. Then, 5 μL of each test solution was spotted onto the surface: Bv-RF2 crude methanol extract (10 mg/mL), surfactin (10 μM, C_53_H_93_N_7_O_13_) (TargetMol, Boston, MA, USA), streptomycin sulfate (0.1 mg/mL), and PBS (control). Plates were incubated at 28 °C for 36–48 h, and inhibition zones were measured using ImageJ (version 1.54f, Wayne Rasband, NIH, Bethesda, MD, USA). Data were analyzed using GraphPad Prism (version 10.1.2, GraphPad Software, San Diego, CA, USA). The experiment was independently repeated three times.

### 2.9. Broad-Spectrum Antimicrobial Activity Assessment

A 10 μL aliquot of Bv-RF2 suspension (OD_600_ = 1.0) was streaked in the center of PDA plates. *Colletotrichum gloeosporioides* or *Alternaria alternata* plugs (8 mm diameter) were placed 15 mm from both sides of the streak in a cross pattern. Control plates contained only fungal plugs. Each treatment included three technical replicates. The experiment was independently repeated three times. After 7 days, inhibition zones were measured, and inhibition rate was calculated as:

Inhibition rate = [(control colony radius − treatment colony radius)/(control colony radius − plug radius)] × 100%.

### 2.10. Statistical Analysis

For assays with technical replicates, technical measurements were first averaged to generate a single value per biological replicate. Data are presented as mean ± standard deviation (SD). Statistical analyses were performed using GraphPad Prism 10.1.2. Unpaired Welch’s *t*-test was used for comparisons between two groups, with Holm–Šidák correction for multiple comparisons. For comparisons involving multiple groups or factors, two-way or one-way analysis of variance (ANOVA) combined with the corresponding post hoc tests was performed. Detailed analytical methods are specified in the legend of each figure. Adjusted *p* values are reported as indicated.

## 3. Results

### 3.1. Isolation and Identification of Phyllosphere Biocontrol Microorganisms

A total of 72 bacterial isolates were recovered from canker-infected citrus leaves and preliminarily identified (Table 1). Using an *Xcc*-GFP-based screening method, strain Bv-RF2 exhibited strong antagonism toward *Xcc*. Under the screening conditions, the fermentation broth of Bv-RF2 achieved an inhibition rate of 78.89%.

### 3.2. Morphological and Phylogenetic Characterization of Bv-RF2

**Figure 1:**
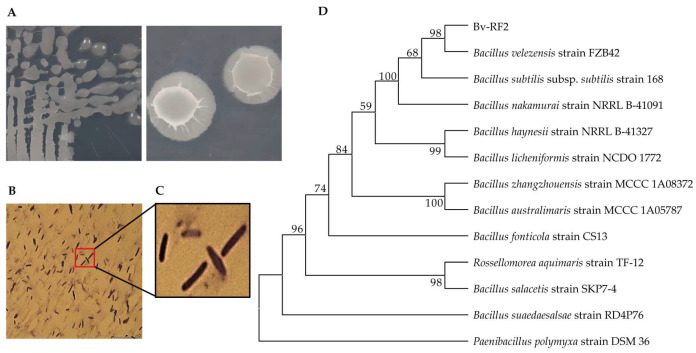
Morphology and Phylogenetic identification of Bv-RF2. (**A**) Colony morphology of Bv-RF2 on NA medium. (**B**) Gram-stained cells observed under 100× objective (scale bar = 250 μm). (**C**) Magnified view of the area shown in (**B**). (**D**) Neighbor-Joining phylogenetic tree based on 16S rRNA gene sequences. The bootstrap consensus tree inferred from 1000 replicates is taken to represent the evolutionary history of the taxa analyzed. Branches corresponding to partitions reproduced in less than 50% bootstrap replicates are collapsed. The percentage of replicate trees in which the associated taxa clustered together in the bootstrap test (1000 replicates) are shown next to the branches. The evolutionary distances were computed using the Maximum Composite Likelihood method and are expressed in the number of base substitutions per site. This analysis involved 13 nucleotide sequences. All positions containing gaps and missing data were eliminated (complete deletion option). There were a total of 1366 positions in the final dataset. Evolutionary analyses were conducted in MEGA 11.

On NA plates, Bv-RF2 formed milky-white colonies with a composite texture, characterized by a dry surface, irregularly wrinkled margins, a viscous interior, and a raised center (Figure 1A). Gram staining revealed purple rod-shaped cells with endospore-like structures (Figure 1B,C), consistent with a Gram-positive *Bacillus* species. The 16S rRNA gene sequence of Bv-RF2 showed 99.8% identity to *Bacillus velezensis* FZB42. Phylogenetic analysis further placed Bv-RF2 within the *B. velezensis* clade with 98% bootstrap support and clearly separated it from *Paenibacillus* spp. (Figure 1D). Based on these results, the strain was designated *Bacillus velezensis* RF2 (Bv-RF2).

### 3.3. Activity and Stability of Bv-RF2 Fermentation Products

**Figure 2:**
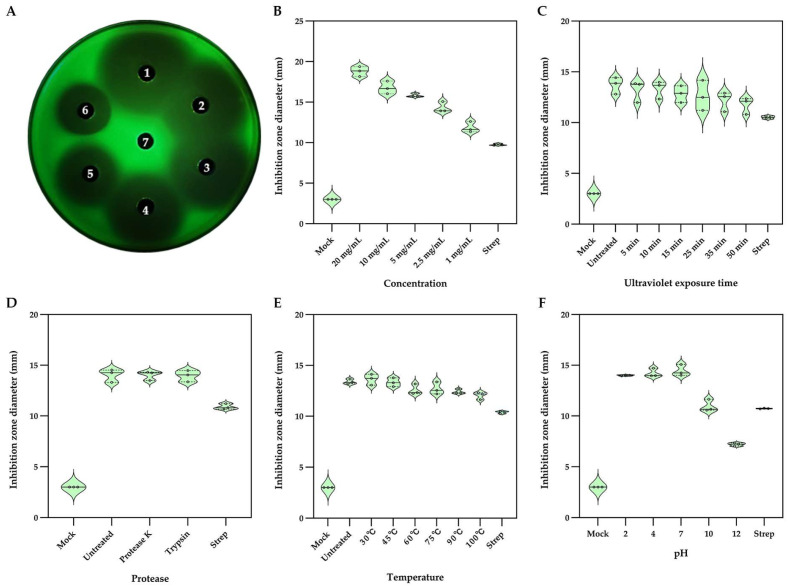
Antagonistic Activity and Stability Analysis of Methanol Extracts of Bv-RF2 against *Xcc* in vitro. (**A**) Inhibition zones formed by methanol extracts at different concentrations against *Xcc*-GFP on NA medium were imaged under LUYOR-3145RG illumination. 1 to 6: the concentrations of the methanol extract: 20 mg/mL, 10 mg/mL, 5 mg/mL, 2.5 mg/mL, 1 mg/mL and streptomycin (Strep, as a positive control, with a working concentration of 0.1 mg/mL), 7: ddH_2_O. (**B**) The corresponding inhibition zone diameters measured by ImageJ. (**C**) Antibacterial stability of methanol extract under different UV lamp irradiation times. (**D**) Antibacterial stability of fermentation broth of methanol extracts treated with protease. (**E**) Antibacterial stability of methanol extracts under different temperature treatments. (**F**) Antibacterial stability of methanol extracts under different pH conditions. The diameter of the agar well is 3 mm. Given the small sample size (n = 3 independent biological replicates), all individual data points are shown; the solid line depicts the median of the three replicates, and the dashed lines denote the quartiles; the violin shape is used only for visualization and not to infer the underlying distribution.

Agar well diffusion assay using the 24 h fermentation broth of Bv-RF2 produced clear inhibition zones against *Xcc*, suggesting that extracellular components in the culture supernatant contributed to the anti-*Xcc* activity. Following acid precipitation and methanol extraction, the crude extract inhibited *Xcc* on NA in a dose-dependent manner (Figure 2A). At 1 mg/mL, the inhibition zone diameter was 11.87 ± 0.55 mm, which was larger than that produced by 0.1 mg/mL streptomycin (9.76 ± 0.09 mm) (Figure 2B).

The antibacterial activity of the crude methanolic extract remained largely stable after UV exposure for 5–50 min, with inhibition zone diameters remaining around 11.76 ± 0.68 mm (Figure 2C). Protease treatment with proteinase K or trypsin did not reduce activity, with the resulting inhibition zone diameters measuring 14.01 ± 0.37 mm and 13.96 ± 0.45 mm, respectively (Figure 2D), and heating at 30–100 °C for 30 min also retained consistent activity, with inhibition zones ≥ 12.03 ± 0.29 mm (Figure 2E). These results are consistent with the involvement of one or more non-proteinaceous and heat-stable bioactive components. At pH 10 and 12, the diameters of the inhibition zones were 10.96 ± 0.49 mm and 7.20 ± 0.13 mm, respectively, compared with 14.45 ± 0.46 mm (untreated, pH 7.0) and 10.72 ± 0.02 mm (0.1 mg/mL streptomycin) (Figure 2F). Although activity decreased under extreme pH conditions, clear inhibition zones were still observed, indicating tolerance to acidic and alkaline conditions.

### 3.4. Genomic and Metabolomic Features of Bv-RF2

**Figure 3:**
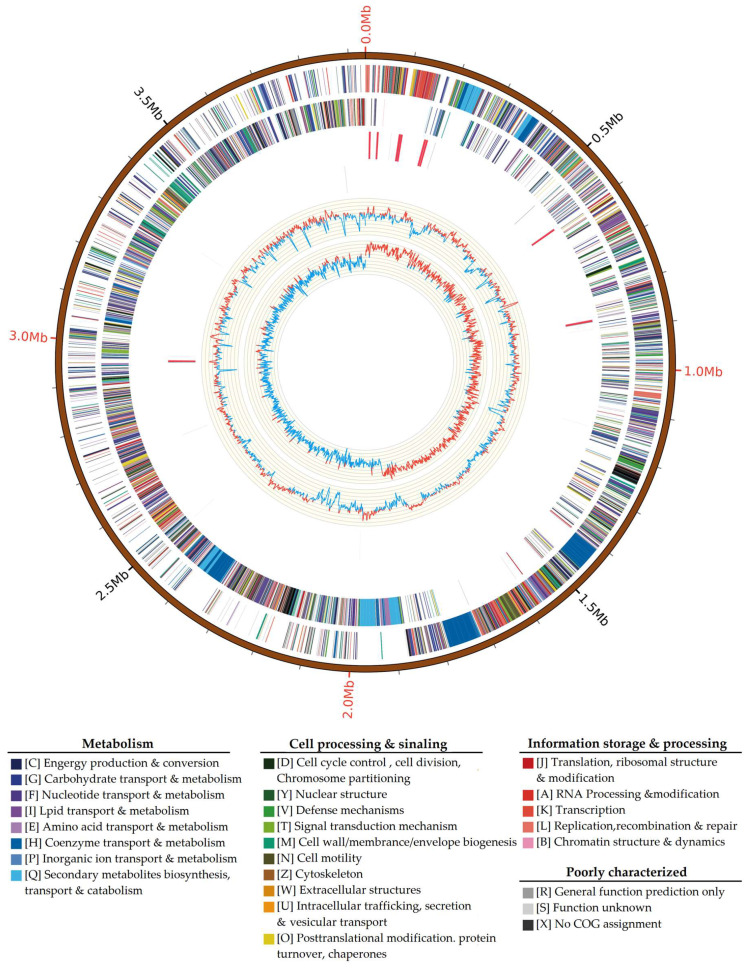
Circular genome map of Bv-RF2. From outer to inner rings: genome coordinates; protein-coding genes on the forward strand; protein-coding genes on the reverse strand; tRNA and rRNA genes on the forward strand; tRNA and rRNA genes on the reverse strand; GC content; and GC skew.

Whole-genome sequencing of Bv-RF2 (GenBank ID: GCA_053847225.1) (Figure 3) revealed a 3,935,256 bp genome containing 3840 predicted genes. antiSMASH analyses identified 12 putative biosynthetic gene clusters (BGCs), including NRPS, transAT-PKS, T3PKS, lanthipeptide, terpene, NRP-metallophore, and other small-molecule clusters. Eight clusters showed high similarity to previously characterized antibiotic or biosurfactant BGCs (e.g., surfactin, fengycin, bacillaene, difficidin, macrolactin H, bacillibactin, and bacilysin), and a locillomycin-like cluster with lower similarity was also predicted (Figure S1). Overall, the BGC repertoire was similar to that of *B. velezensis* FZB42 (GenBank ID: GCA_000015785.2). Notably, several BGCs associated with lipopeptide biosynthesis (e.g., surfactin) were present at the genomic level, whereas the corresponding metabolites were not detected in our untargeted metabolomic analysis, partly because their *m*/*z* values may fall outside the acquired mass range of the LC–MS/MS method used in this study (Table S2).

In addition to secondary metabolism, the genome encoded 162 carbohydrate-active enzymes, six putative resistance genes, 841 predicted transmembrane proteins, and 41 predicted secreted proteins, consistent with a capacity for nutrient acquisition, environmental adaptation, and secretion of extracellular factors.

### 3.5. Bv-RF2 Elicits Defense Priming in Sweet Orange

**Figure 4:**
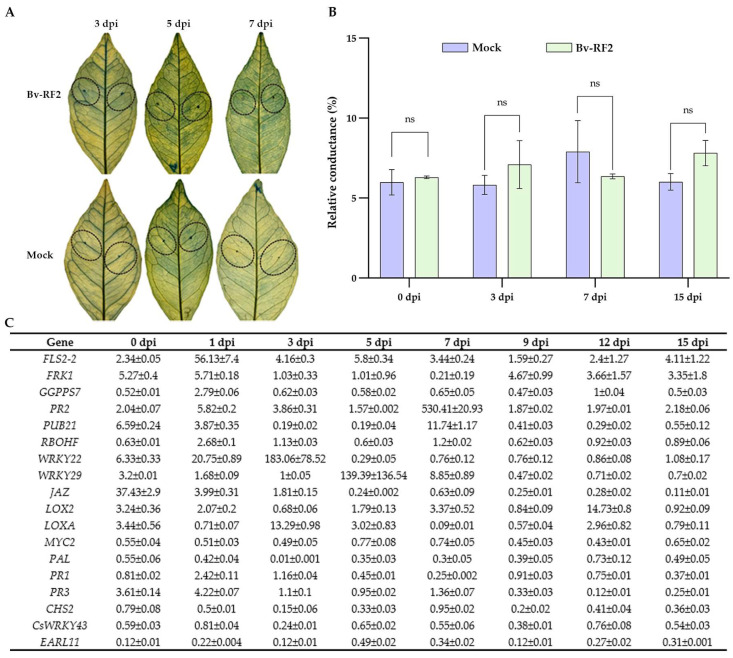
Physiological responses and defense-related gene expression in sweet orange leaves after Bv-RF2 infiltration. (**A**) Evans blue staining at the indicated time points. The dashed circle indicates the infiltration area. (**B**) Relative electrolyte leakage at the indicated time points. Data are shown as mean ± SD (n = 3 independent biological replicates). Statistical significance between Mock and Bv-RF2 at each time point was assessed using ordinary two-way ANOVA followed by Sidak’s multiple comparisons test. Adjusted *p* values are reported. Statistical significance: ns, not significant (*p* > 0.05). (**C**) RT–qPCR analysis of defense-related genes from 0 to 15 dpi. Relative expression was calculated using the 2^−ΔΔCt^ method, and data are shown as mean ± SD (n = 3 independent biological replicates).

To assess plant responses to Bv-RF2, physiological changes and defense-related gene expression were monitored after leaf infiltration. Evans blue staining showed only slight color intensification between 3 and 15 dpi (Figure 4A), indicating limited cell death. Conductivity measurements revealed a transient increase followed by stabilization (Figure 4B), suggesting mild and reversible changes in membrane permeability.

RT-qPCR analysis revealed a transient induction pattern of several defense-related genes (Figure 4C), and statistical analyses of the corresponding adjusted *p* values are provided in Table S3. *PR2* transcripts increased at 3 dpi, peaked at 7 dpi, and declined by 12 dpi. *PR1* and *PR3* were also upregulated at 3–7 dpi. Genes associated with PAMP-triggered immunity (PTI), including *FLS2-2* and *RBOHF*, were upregulated by 56-fold and 2.7-fold, respectively, at 3 dpi, consistent with a modest PTI-like response. *WRKY22* and *WRKY29* were induced from 3 to 12 dpi but at levels lower than typically observed during infection with virulent pathogens, suggesting a priming-associated defense state rather than strong defense activation. Several JA pathway-associated genes (e.g., *JAZ*, *LOX2*, and *LOXA*) exhibited dynamic expression changes, and *CHS2* and *PAL* were upregulated at 3–7 dpi, consistent with enhanced phenylpropanoid-related responses.

### 3.6. Biocontrol Efficacy of Bv-RF2 Against Citrus Canker

**Figure 5:**
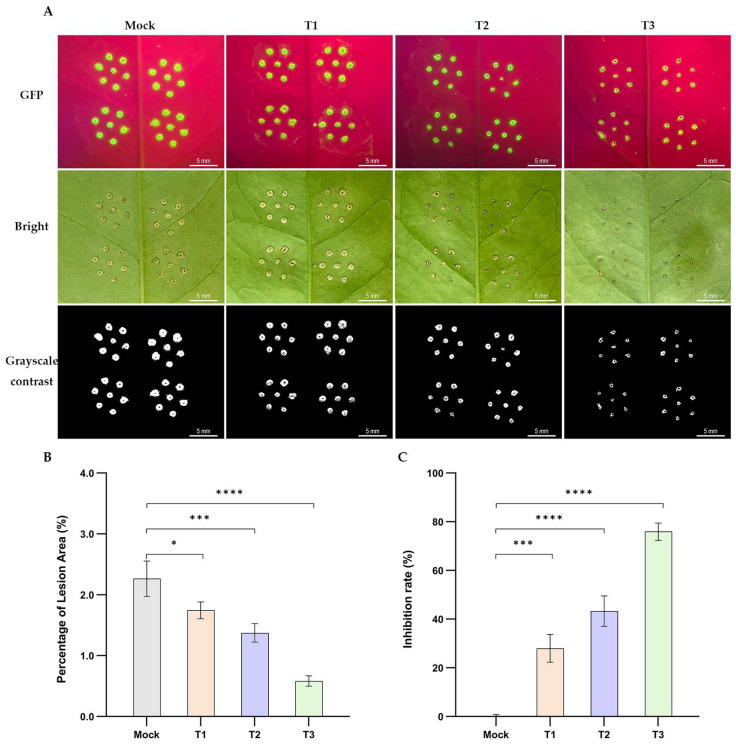
In planta activity of Bv-RF2 crude extracts against citrus canker. (**A**) Representative symptoms at 7 dpi on sweet orange leaves inoculated with *Xcc*-GFP mixed with Bv-RF2 extracts (1, 5, or 10 mg/mL). GFP fluorescence images were acquired using LUYOR-3145RG illumination. (**B**) Quantification of lesion area at 7 dpi. (**C**) Disease inhibition rate calculated from lesion area. Data are shown as mean ± SD (n = 3 independent biological replicates). Statistical analysis was performed using Ordinary one-way ANOVA followed by Dunnett’s multiple comparisons test, and adjusted *p* values are reported. Statistical significance: * *p* < 0.05, *** *p* < 0.001, **** *p* < 0.0001.

Needle-inoculation assays were performed to evaluate whether Bv-RF2 crude methanolic extracts could reduce canker development caused by *Xcc* on sweet orange leaves. Co-inoculation of *Xcc* with increasing concentrations of the extract resulted in a clear, dose-dependent reduction in lesion area (Figure 5A,B). Quantitatively, extracts at 10, 5, and 1 mg/mL reduced disease severity by 75.93%, 43.32%, and 27.98% (Figure 5C). These results indicate that Bv-RF2-derived soluble components possess in planta activity under our assay conditions.

### 3.7. Broad-Spectrum Antimicrobial Activity of Bv-RF2

**Figure 6:**
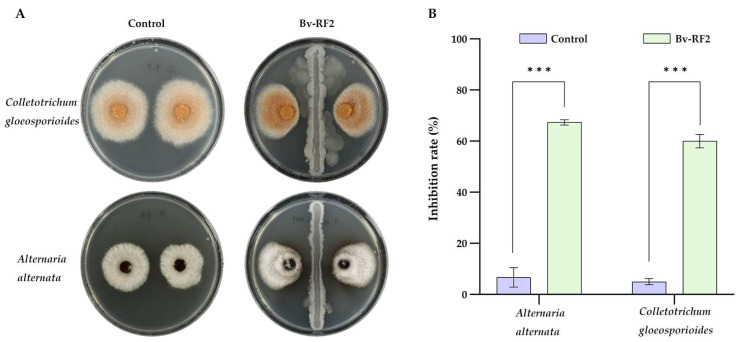
Broad-spectrum inhibitory activity of Bv-RF2 against citrus fungal pathogens in vitro. (**A**) Representative images showing the inhibition of mycelial growth of *Colletotrichum gloeosporioides* and *Alternaria alternata* by Bv-RF2 on PDA plates. (**B**) Quantification of the inhibition rate of mycelial growth. Data are shown as mean ± SD (n = 3 independent biological replicates). Statistical analysis was performed using unpaired Welch’s *t* tests with Holm–Šidák correction for multiple comparisons, and adjusted *p* values are reported. Statistical significance: *** *p* < 0.001.

Bv-RF2 also showed inhibitory activity against fungal pathogens in vitro. On PDA plates, Bv-RF2 effectively inhibited the mycelial growth of *Colletotrichum gloeosporioides* and *Alternaria alternata* (Figure 6A), with inhibition rates of 60.02% and 67.34% (Figure 6B). Together with the anti-*Xcc* results, these data suggest that Bv-RF2 exhibits broad inhibitory potential against multiple citrus-associated pathogens under the tested conditions.

## 4. Discussion

Guided by the “Cry for Help” hypothesis, we used the phyllosphere microbiome of *Xcc*-infected citrus as a targeted resource for biocontrol screening. Plants under biotic or abiotic stress can actively modulate their associated microbiota via changes in exudation patterns, thereby recruiting beneficial microbes that enhance stress tolerance and disease resistance [52,53]. As the phyllosphere is the primary infection site for CBC, pathogen-induced changes in leaf exudates and volatile organic compounds (VOCs) may reshape microbial communities and enrich stress-tolerant antagonists capable of producing inhibitory factors and/or competing for epiphytic niches [22,35,54]. Consistent with this framework, our *Xcc*-GFP-based screening enabled efficient identification of an adapted antagonist, Bv-RF2.

Bv-RF2 exhibited inhibitory activity against *Xcc* and suppressed the growth of *Colletotrichum gloeosporioides* and *Alternaria alternata* in vitro. *Bacillus* spp., and *B. velezensis* in particular, are known to produce a broad arsenal of antimicrobial metabolites, including lipopeptides, polyketides, and ribosomally and non-ribosomally synthesized peptides that contribute to biocontrol [55,56,57]. The crude extract displayed stability to heat, protease treatment, and a broad pH range (Figure 2), which is consistent with the involvement of non-proteinaceous, chemically stable bioactive components reported for *B. velezensis* [55,56,58]. Importantly, our in planta assays were conducted using crude methanolic extracts to primarily assess the contribution of soluble/secreted components while minimizing variability introduced by foliar colonization dynamics. We acknowledge that practical field-level biocontrol efficacy depends not only on antimicrobial metabolites but also on traits such as epiphytic colonization, persistence, and competitive fitness on leaf surfaces [59]. Our preliminary results indicate that this strain can be recruited into leaf tissues following pathogen challenge. Moreover, Evans blue staining further suggests good host compatibility and biocontrol potential, implying that the strain may be able to persist and/or stably colonize host leaf tissues. Therefore, the extract-based assays provide evidence for metabolite-associated activity but do not represent a complete evaluation of live-bacterium biocontrol performance. The practical efficacy under field-relevant conditions still needs to be validated through foliar application trials using live bacteria, together with quantitative assessments of leaf-surface attachment and colonization, persistence, and competitive fitness.

Genome mining revealed that Bv-RF2 harbors multiple predicted BGCs with similarity to clusters involved in the biosynthesis of lipopeptides and polyketides (e.g., surfactin, fengycin, difficidin, and macrolactin) [57]. However, several genome-predicted products were not detected in our untargeted metabolomic analysis, which may reflect limitations of the acquired *m/z* range and/or other dataset constraints (Table S2). Thus, BGC predictions should be interpreted as generating mechanistic hypotheses rather than direct proof that specific metabolites mediate the observed phenotypes. Establishing causal genotype-phenotype links will require targeted LC-MS/MS analyses, bioassay-guided fractionation, and genetic perturbation approaches [55,56].

Regarding host responses, Bv-RF2 infiltration caused limited cell death and mild, reversible membrane changes (Figure 4A,B). The transient induction of defense-related genes, together with moderate activation of PTI-associated markers and phenylpropanoid-related genes (Figure 4C), is more consistent with an ISR-like response and defense priming than with strong systemic defense activation typically induced by virulent pathogens [53,60,61]. Such priming may sensitize host defenses and contribute to reduced disease development upon pathogen challenge, while potentially avoiding severe fitness costs.

Overall, our data support two complementary and testable hypotheses for Bv-RF2-associated protection: (i) secretion of chemically stable bioactive components with antimicrobial activity, and (ii) elicitation of ISR-like priming in the host. These findings provide a foundation for further mechanistic validation and for developing environmentally friendly strategies for CBC management.

## 5. Conclusions

In conclusion, we identified *Bacillus velezensis* RF2 (Bv-RF2) from the phyllosphere of *Xcc*-infected citrus and demonstrated its inhibitory activity against *Xcc* as well as other citrus-associated pathogens under our experimental conditions. Crude methanolic extracts exhibited strong antibacterial activity in vitro and reduced canker development in a leaf assay, indicating that Bv-RF2-derived soluble components can contribute to disease suppression. Genome mining revealed a repertoire of predicted NRPS/PKS-type BGCs that is consistent with the capacity to produce diverse secondary metabolites; however, causal links between specific clusters/metabolites and antimicrobial activity require further targeted validation. Bv-RF2 infiltration was associated with an ISR-like, priming-related host response characterized by transient induction of defense-related genes. Future work should evaluate live-bacterium foliar biocontrol performance, including colonization and persistence, and apply targeted chemical and genetic approaches to strengthen genotype–phenotype relationships.

## Figures and Tables

**Table 1:** Information on Strain Isolation and Identification.

Strain No.	Strain Name	Genus	Source	Distribution
1 (Bv-RF2)	Bw-E1-2 1 L	*Bacillus*	*Citrus sinensis*	leaf endosphere
2	Ae-5-2-3	*Burkholderia*	*Citrus sinensis*	phyllosphere
3	Bw-E2-4 R	*Xanthomonas*	*Citrus sinensis*	leaf endosphere
4	Bw-R2-4 R	*Curtobacterium*	*Citrus sinensis*	phyllosphere
5	Bw-R2-3 R	*Sphingomonas*	*Citrus sinensis*	phyllosphere
6	Bw-E-6 R	*Atlantibacter*	*Citrus sinensis*	leaf endosphere
7	Bw-R2-2 R	*Sphingomonas*	*Citrus sinensis*	phyllosphere
8	Bw-E-3 R	*Xanthomonas*	*Citrus sinensis*	leaf endosphere
9	Bw-E-1 R	*Sphingomonas*	*Citrus sinensis*	leaf endosphere
10	Bw-E-2 R	*Sphingomonas*	*Citrus sinensis*	leaf endosphere
11	Bw-R2-6 1 R	*Curtobacterium*	*Citrus sinensis*	phyllosphere
12	Bv-E2-1 K	*Paenibacillus*	*Citrus sinensis*	leaf endosphere
13	Bw-R2-2 1 K	*Brevundimonas*	*Citrus sinensis*	phyllosphere
14	Bu-E2-3 K	*Atlantibacter*	*Citrus sinensis*	leaf endosphere
15	Bu-R2-1 T	*Pseudomonas*	*Citrus sinensis*	phyllosphere
16	Bw-R1-2 T	*Curtobacterium*	*Citrus sinensis*	phyllosphere
17	Bw-R2-2 T	*Curtobacterium*	*Citrus sinensis*	phyllosphere
18	Bw-R2-3 T	*Curtobacterium*	*Citrus sinensis*	phyllosphere
19	Bv-E1-2 2 K	*Atlantibacter*	*Citrus sinensis*	leaf endosphere
20	Bu-R1-2 t	*Atlantibacter*	*Citrus sinensis*	phyllosphere
21	Bv-E2-3 t	*Pseudomonas*	*Citrus sinensis*	leaf endosphere
22	Bw-R2-1 t	*Pseudomonas*	*Citrus sinensis*	phyllosphere
23	Bw-R2-2 t	*Niallia*	*Citrus sinensis*	phyllosphere
24	Bv-E1-3 t	*Atlantibacter*	*Citrus sinensis*	phyllosphere
25	Bu-R2-2-2 t	*Atlantibacter*	*Citrus sinensis*	phyllosphere
26	Bu-R1-1 t	*Atlantibacter*	*Citrus sinensis*	phyllosphere
27	Bu-E2-2 t	*Pseudomonas*	*Citrus sinensis*	leaf endosphere
28	Bu-E2-5 t	*Pseudomonas*	*Citrus sinensis*	leaf endosphere
29	Bu-E1-1 t	*Atlantibacter*	*Citrus sinensis*	leaf endosphere
30	Bu-E1-2 t	*Atlantibacter*	*Citrus sinensis*	leaf endosphere
31	Bu-R2-2-1 K	*Pseudomonas*	*Citrus sinensis*	phyllosphere
32	Bu-R2-2-2 K	*Atlantibacter*	*Citrus sinensis*	phyllosphere
33	Bu-R2-3 K	*Atlantibacter*	*Citrus sinensis*	phyllosphere
34	Bw-R1-3 K	*Stenotrophomonas*	*Citrus sinensis*	phyllosphere
35	Bu-E1-1 K	*Pseudomonas*	*Citrus sinensis*	leaf endosphere
36	Bu-R2-1 K	*Atlantibacter*	*Citrus sinensis*	phyllosphere
37	Bw-R1-1 K	*Methylobacterium*	*Citrus sinensis*	phyllosphere
38	Bv-E2-1 N	*Atlantibacter*	*Citrus sinensis*	leaf endosphere
39	Bw-R1-1 t	*Curtobacterium*	*Citrus sinensis*	phyllosphere
40	Bu-E1-3 N	*Atlantibacter*	*Citrus sinensis*	leaf endosphere
41	Bw-R1-4 K	*Agrobacterium*	*Citrus sinensis*	phyllosphere
42	Bu-R2-2 N	*Pseudomonas*	*Citrus sinensis*	phyllosphere
43	Bw-R2-4 N	*Stenotrophomonas*	*Citrus sinensis*	phyllosphere
44	Bu-E1-2 N	*Pseudomonas*	*Citrus sinensis*	leaf endosphere
45	Bu-E1-2-1 T	*Atlantibacter*	*Citrus sinensis*	leaf endosphere
46	Bw-E1-3 K	*Allohumibacter*	*Citrus sinensis*	leaf endosphere
47	Bw-R1-4 2 K	*Agrobacterium*	*Citrus sinensis*	phyllosphere
48	Bw-R2-3 N	*Achromobacter*	*Citrus sinensis*	phyllosphere
49	Bw-E1-1 1 L	*Atlantibacter*	*Citrus sinensis*	leaf endosphere
50	Bu-E2-1 L	*Atlantibacter*	*Citrus sinensis*	leaf endosphere
51	Bw-R2-1 L	*Mammaliicoccus*	*Citrus sinensis*	phyllosphere
52	Bw-E1-2 2 L	*Burkholderia*	*Citrus sinensis*	leaf endosphere
53	Bv-E2-2 L	*Atlantibacter*	*Citrus sinensis*	leaf endosphere
54	Bu-R2-3 L	*Atlantibacter*	*Citrus sinensis*	phyllosphere
55	Bw-R1-2 L	*Microbacterium*	*Citrus sinensis*	phyllosphere
56	Bv-E1-1 t-T	*Pseudomonas*	*Citrus sinensis*	leaf endosphere
57	Bw-E2-2 L	*Priestia megaterium*	*Citrus sinensis*	leaf endosphere
58	Bw-R1-3-1 L	*Staphylococcus*	*Citrus sinensis*	phyllosphere
59	Bv-E2-1 T	*Pseudomonas*	*Citrus sinensis*	leaf endosphere
60	Bw-R1-1 1 T	*Stenotrophomonas*	*Citrus sinensis*	phyllosphere
61	Bv-E2-3 T	*Stenotrophomonas*	*Citrus sinensis*	leaf endosphere
62	Bw-E-2 R-L	*Sphingomonas*	*Citrus sinensis*	leaf endosphere
63	Bw-R2-6 R-L	*Curtobacterium*	*Citrus sinensis*	phyllosphere
64	Bw-E-2 L	*Sphingomonas*	*Citrus sinensis*	leaf endosphere
65	Bw-R2-6 L	*Curtobacterium*	*Citrus sinensis*	phyllosphere
66	Bv-E2-2 T	*Pseudomonas*	*Citrus sinensis*	leaf endosphere
67	Bu-E1-1 1 N	*Pseudomonas*	*Citrus sinensis*	leaf endosphere
68	Bu-E2-1 N	*Pseudomonas*	*Citrus sinensis*	leaf endosphere
69	Bu-E1-2 T	*Atlantibacter*	*Citrus sinensis*	leaf endosphere
70	Bw-R2-1 1 R	*Rhodococcus*	*Citrus sinensis*	phyllosphere
71	Bw-E2-4 1 N	*Xanthomonas*	*Citrus sinensis*	leaf endosphere
72	Bu-E1-2 K	*Pseudomonas*	*Citrus sinensis*	leaf endosphere

Note: All strains listed in the table were isolated from *Citrus sinensis* (L.) Osbeck leaves infected with *Xcc*. The sampling date was 14 July 2019, and the sampling location was Ganxian, Ganzhou, China.

## Data Availability

The original contributions presented in this study are included in the article/Supplementary Materials. Further inquiries can be directed to the corresponding authors.

## Supplementary Materials

The following supporting information can be downloaded at: https://www.mdpi.com/article/10.3390/microorganisms14010121/s1, Table S1. qPCR Primer Information. Table S2. Bv-RF2 untargeted metabolomics data. Table S3. RT-qPCR analysis of defense-related genes from 0 to 15 dpi. Figure S1. NRPS and PKS Biosynthetic Loci of the Bv-RF2 Genome. Biosynthetic loci identified by antiSMASH from the Bv-RF2 genome that contained NRPS and PKS of core biosynthetic genes. Predictions of the organization of the biosynthetic domains in each locus shown here were determined by MIBiG. Full names for the biosynthetic domains are given in the right table. References [62,63,64] are cited in the supplementary materials.
