# Supplementary material for: Bacillus velezensis RF2 Rescued from Citrus Phyllosphere: Dual Mechanisms and Broad-Spectrum Activity for Controlling Citrus Bacterial Canker
Source: Microorganisms. 2026 Jan 6;14(1):121. doi: 10.3390/microorganisms14010121 (PMC12843974; doi:10.3390/microorganisms14010121)
Supplement: Supplementary file 1 [file microorganisms-14-00121-s001.zip › microorganisms-4050731-supplementary/Supplementary Materials/Table S1 and Figure S1.pdf]

Supplementary Materials:

Table S1. qPCR Primer Information.

Gene Name	Forward (5′-3′)	Reverse (5′-3′)	
CsGAPDH	GGAAGGTCAAGATCGGAATCAA	CGTCCCTCTGCAAGATGACTCT	References **Error! Reference source not found.**
CHS2	CACTGTTCTTCGCCTCGCTA	AGCTGATACAACGGACGCTC	References **Error! Reference source not found.**
CsWRKY43	AGTCCACAGATTGGTGTTGC	TTTCTTGGCCATGCAACTGC	References **Error! Reference source not found.**
EARL11	TGTCACTATTGGCACACCCC	GGCTGAGTGAAAGCGGGATA	References **Error! Reference source not found.**
FRK1	AGTGACGTGTACAGCTTTGG	TCTGATATTTCCCTCGGCAAG	References **Error! Reference source not found.**
GGPPS7	AGCGTGTGCGTCCTCTTTTA	CTTTGACCGTCTCCGCTCAT	References **Error! Reference source not found.**
JAZ	ATGTCGAGCTCAGGCCAAAA	TGCTCCGTTCTGGCTTCATT	References **Error! Reference source not found.**
LOX2	GAGGAAGCTCGAACACACGA	GAGCTTTGGCTAGCCTCCAA	References **Error! Reference source not found.**
LOXA	GTGACGAGGTCAACAGGGTT	GCAATGACGAATGGCTCGAC	References **Error! Reference source not found.**
MYC2	GCGAGCTGTTGTGCCTAATG	GCCAACTCCTTCTTCACCGA	References **Error! Reference source not found.**
PAL	TCCCCTTTTCGTACATCGCC	ACGCAAGTCCCTCTTTAGGC	References **Error! Reference source not found.**
PR1	AAATGTGGGTGAATGAGAAAGC	ATTATTGTTGCACGTCACCTTG	References **Error! Reference source not found.**
PR2	TTCCACTGCCATCGAAACTG	GTAATCTTGTTTAAATGAGCCTCTTG	References **Error! Reference source not found.**
PR3	GGCTCAAACTTCACATGAAACTAC	GTTGACAATAATCTCCAGGGTTTC	References **Error! Reference source**

			**not found.**
PUB21	CGTTGGTCGTCGTCTATCGT	AATGGAGACTGCGAACTCCG	References **Error! Reference source not found.**
RBOHF	AAGCAGTGGCAAAAGCGAAG	TCAAAGCTCGAGCCGCTAAA	References **Error! Reference source not found.**
WRKY22	AGGAAACAGGTGGAACGAAG	GACCTTGAGCCTTTGACATTG	References **Error! Reference source not found.**
WRKY29	ACATATACGGCAGAGCACAAC	AGTGGGTTTGGTTGAGGAAG	References **Error! Reference source not found.**

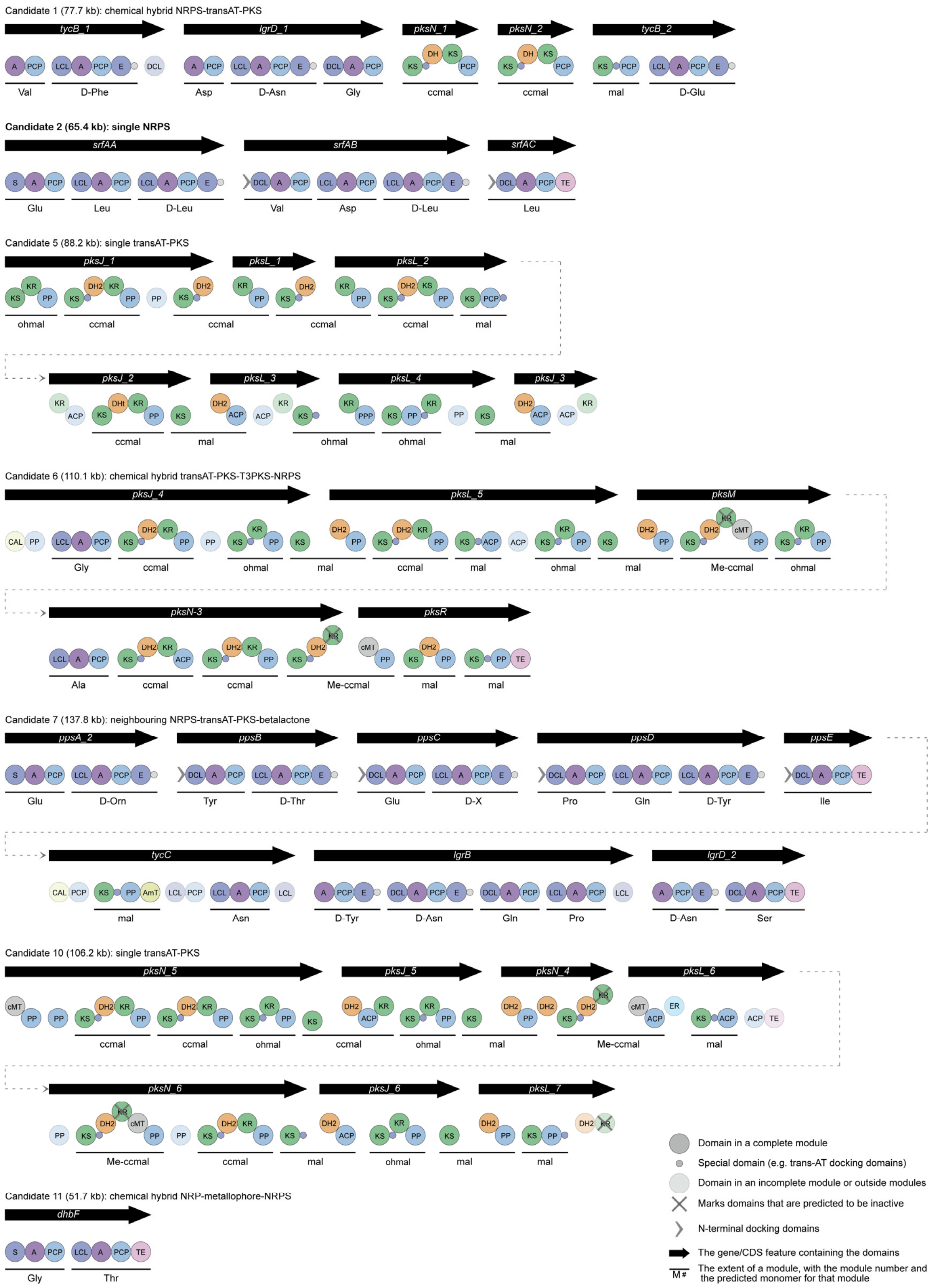

Figure S1. NRPS and PKS Biosynthetic Loci of the Bv-RF2 Genome. Biosynthetic loci identified by antiSMASH from the Bv-RF2 genome that contained NRPS and PKS of core biosynthetic genes. Predictions of the organization of the biosynthetic domains in each locus shown here were determined by MIBiG. Full names for the biosynthetic domains are given in the right table.
